# Leadership by Nurse Managers and its Impact on Humanization. A Descriptive Analysis based on the Perception of Care Nurses

**DOI:** 10.17533/udea.iee.v43n3e03

**Published:** 2025-10-17

**Authors:** Alberto Cortés Borra, Pedro Jaén Ferrer, Vicente Gea-Caballero, Sandra Martín García, Gemma Martínez Estalella, José Ramón Martínez Riera

**Affiliations:** 1 Nurse, M.Sc. Ph.D. candidate. Universidad de Alicante, Spain. Email: alcortesborra@gmail.com. Corresponding author. https://orcid.org/0000-0002-4809-8364 Universidad de Alicante Universidad de Alicante Spain alcortesborra@gmail.com; 2 Nurse, M.Sc. Coach personal, La Manga del Mar Menor, Spain. Email: pjaenf@gmail.com. https://orcid.org/0009-0002-2752-1853 HUGES - Humanization of management La Manga del Mar Menor Spain pjaenf@gmail.com; 3 Nurse, Ph. D. Faculty of Health Sciences, Research Group SALCOM in Community Health and Care, Valencian International University, Valencia, Spain. Email: vagea@universidadviu.com. https://orcid.org/0000-0001-8607-3195 Valencian International University Faculty of Health Sciences Research Group SALCOM in Community Health and Care Valencian International University Valencia Spain vagea@universidadviu.com; 4 Ph.D. Professor at ESIC University. Spain. Email: sandra@people4.es. https://orcid.org/0009-0000-8305-8176 ESIC University ESIC University Spain sandra@people4.es; 5 Nurse, M.Sc. Deputy of Strategy and Planning Direction Hospital Clinic of Barcelona. Associate Professor University of Barcelona. Spain. Email: GEMMA@clinic.cat. https://orcid.org/0000-0002-5742-8919 Universitat de Barcelona Deputy of Strategy and Planning Direction Hospital Clinic of Barcelona University of Barcelona Spain GEMMA@clinic.cat; 6 Nurse, M.Sc. Ph.D. Honorable Professor at Departamento Enfermería Comunitaria, Medicina Preventiva y Salud Pública; Universidad de Alicante, Spain. Email: josera.ferranna@gmail.com. https://orcid.org/0000-0002-4926-6622 Universidad de Alicante Departamento Enfermería Comunitaria, Medicina Preventiva y Salud Pública Universidad de Alicante Spain josera.ferranna@gmail.com; 7 Asociación de Enfermería Comunitaria, Grupo de Humanización, Spain. Asociación de Enfermería Comunitaria Asociación de Enfermería Comunitaria Grupo de Humanización Spain; 8 HUGES - Humanization of management, Spain. HUGES HUGES - Humanization of management Spain

**Keywords:** nursing, humanization of assistance, leadership, health management, latent class analysis, primary care nursing., enfermería, liderazgo, humanización de la atención, gestión en salud, análisis de clases latentes, enfermería de atención primaria., enfermagem, liderança, humanização do cuidado, gestão em saúde, análise de classes latentes, enfermagem de atenção primária.

## Abstract

**Objective.:**

The objective was to analyze the relationship among sociodemographic factors, management variables, and the perception of care humanization by nursing staff.

**Methods.:**

A descriptive and observational study was conducted with a sample of 659 nurses working in Spain. A descriptive analysis of the sociodemographic and management variables was performed. Subsequently, a Latent Class Analysis (LCA) was applied to classify participants according to their scores on the HUMAS scale, which measures their perception of their own humanization and that of their manager. Finally, multinomial and logistic regression models were proposed to identify the determinant factors for belonging to these classes and for the opinion on the center of the healthcare system.

**Results.:**

The sample was composed mostly of women (78.9%) with professional experience and job stability averaging 24.17±10.49 years (Min=1, Max=45). Notable dissatisfaction (58.3%) and distrust (57.9% between little and none) towards managers were detected. The LCA identified four nurse profiles based on their perception of humanization: a class with a high valuation of their own humanization and management (Class 2: c2), one with a low valuation in both (Class 3: c3), one with a high personal valuation but low towards management (Class 4: c4), and an intermediate class (Class 1: c1). The most valued traits in a manager were respect, communication, and transparency.

**Conclusion.:**

A significant gap exists between nurses’ self-perception of humanization, which is generally high, and their perception of their managers, which is predominantly low. This dissonance, combined with dissatisfaction with leadership, underscores the need to develop management styles that promote communication, respect, and transparency to improve the work environment and the quality of patient care.

## Resumen

**Objetivo.:**

Analizar la relación entre factores sociodemográficos, variables de gestión y la percepción de la humanización del cuidado por parte del personal de enfermería.

**Métodos.:**

Se realizó un estudio descriptivo y observacional con una muestra de 659 enfermeras que trabajan en España. Se llevó a cabo un análisis descriptivo de las variables sociodemográficas y de gestión. Posteriormente, se aplicó un Análisis de Clases Latentes (LCA) para clasificar a las participantes según sus puntuaciones en la escala HUMAS, que mide la percepción de la humanización propia y la de su gestor. Finalmente, se propusieron modelos de regresión multinomial y logística para identificar los factores determinantes de la pertenencia a dichas clases y de la opinión sobre el centro del sistema sanitario.

**Resultados.:**

La muestra estuvo compuesta mayoritariamente por mujeres (78,9 %) con una experiencia profesional y estabilidad laboral con un promedio 24,17±10,49 DE años (Mín = 1, Max = 45). Se detectó una notable insatisfacción (58,3 %) y desconfianza (57,9 % entre poca y ninguna) hacia los gestores. El LCA identificó cuatro perfiles de enfermeras según su percepción de la humanización: una clase con alta valoración de la humanización propia y de la gestión (Clase 2: c2), una con baja valoración en ambas (Clase 3: c3), una con alta valoración personal pero baja hacia la gestión (Clase 4: c4) y una clase intermedia (Clase 1: c1). Los rasgos más valorados en un gestor fueron el respeto, la comunicación y la transparencia.

**Conclusión.:**

Existe una brecha significativa entre la autopercepción de humanización de las enfermeras, generalmente alta, y la percepción que tienen de sus gestores, que es predominantemente baja. Esta disonancia, unida a la insatisfacción con el liderazgo, subraya la necesidad de desarrollar estilos de gestión que promuevan la comunicación, el respeto y la transparencia para mejorar tanto el entorno laboral como la calidad de la atención al paciente.

## Resumo

**Objetivo.:**

analisar a relação entre fatores sociodemográficos, variáveis de gestão e a percepção da equipe de enfermagem sobre a humanização do cuidado.

**Métodos.:**

Foi realizado um estudo descritivo e observacional com uma amostra de 659 enfermeiros da Espanha. Foi realizada uma análise descritiva das variáveis sociodemográficas e de gestão. Subsequentemente, foi aplicada uma Análise de Classes Latentes (ACL) para classificar os participantes de acordo com suas pontuações na escala HUMAS, que mede sua percepção de sua própria humanização e a de seu gestor. Finalmente, foram propostos modelos de regressão multinomial e logística para identificar os fatores determinantes para a adesão a essas classes e para as opiniões sobre o centro do sistema de saúde.

**Resultados.:**

A amostra consistiu principalmente de mulheres (78.9%) com ampla experiência profissional e estabilidade no emprego. Foi detectada uma insatisfação significativa (58.3%) e desconfiança (57.9% entre pouco e nenhum) em relação aos gestores. A LCA identificou quatro perfis de enfermeiros com base em sua percepção de humanização: uma classe com alta valorização da própria humanização e a do gerente (Classe 2: c2), uma com baixa valorização em ambas (Classe 3: c3), uma com alta valorização pessoal, mas baixa em relação à gestão (Classe 4: c4), e uma classe intermediária (classe 1: c1). As características mais valorizadas em um gerente eram respeito, comunicação e transparência.

**Conclusão.:**

Existe uma lacuna significativa entre a auto-percepção dos enfermeiros sobre a humanização, que é geralmente alta, e a percepção deles sobre seus gestores, que é predominantemente baixa. Essa dissonância, juntamente com a insatisfação com a liderança, destaca a necessidade de desenvolver estilos de gestão que promovam a comunicação, o respeito e a transparência para melhorar tanto o ambiente de trabalho quanto a qualidade do atendimento ao paciente.

## Introduction

Modern healthcare is always in the search for the balance among effectiveness, technological efficiency, and the need to preserve and enhance the human dimension of care. In this important equilibrium, humanization is not configured as a mere desirable attribute, rather, it must be a fundamental pillar of the care quality, especially evident for the nursing profession, given that it is at the forefront of direct and continuous contact with patients, families and community, with nurses having the unique ability to shape the care experience. Nevertheless, the development of truly humanized care by nurses is not an individual act; it is intrinsically conditioned by organizational structures, prevailing leadership styles, and management dynamics that articulate health systems.^1^

The concept of humanization in health transcends the mere application of techniques and procedures; it implies recovering the essence of care, which places individuals as a whole (with their physical, emotional, social and spiritual dimensions) at the center of attention, beyond their pathologies or other conditionings.^2^ Despite it all, this process cannot be separated from the wellbeing and professional development of those who provide it. The concept of "professional-centrism", far from being an antonym of “patient-centrism”, emerges as a paradigm shift to ensure sustainable, high-quality patient care.^3^ A work environment that is not humanized for the professional runs the risk of indirectly dehumanizing the care provided to patients.^4^ This interdependence generates a core ethical dilemma in healthcare management: How can the patient’s undisputed priority be harmonized with the imperative need to care for those who care?

Due to this, leadership and nursing management acquire a fundamental relevance as leaders of a humane care environment,^5^^-^^7^ the work environment, staff motivation, levels of professional satisfaction and - lastly - the perceived quality of care.^8^^,^^9^ Leadership that genuinely promotes empathy, effective communication, mutual respect, and transparency may not only significantly enhance the degree of humanization in care, but can also mitigate the adverse effects of a system under pressure from demands. On the contrary, more authoritarian, depersonalized, or productivity-focused management approaches could wear down team morale and seriously compromise the ability to provide humane care.^10^

Leadership styles based on emotional intelligence, made popular by Daniel Goleman,^11^^-^^12^ provide an exceptionally pertinent conceptual framework to understand how leaders can be catalysts for the humanization of care. Goleman identifies styles, such as affiliative, democratic, and coaching, which promote empathy, collaboration, and personal development - characteristics of leadership that actively seeks to promote humanization.^3^^-^^12^ Applying these styles improves the leader-team relationship and is transmitted to the professional-patient relationship.^13^

This article analyzes the perception of humanization, leadership, and management by a sample of nursing professionals in Spain, exploring how sociodemographic and occupational factors, as well as the characteristics and styles of their managers, relate to the perceived level of humanization. The study used the HUMAS scale, a validated instrument^14^ that permits a structured measurement of humanization in nursing care, both from the professionals’ self-perceptions and from their managers’ perceptions.^8^ Furthermore, the work examines the complex vision of nurses on the equilibrium between patients and professionals as the core of the health system. Finally, direct connections will be established between the empirical findings and Goleman’s leadership styles,^11^^,^^12^ to propose specific recommendations for effective, humane, and ethically sound nursing management. The aim of this study was to explore the perceptions of humanization, leadership, and management from the perspectives of nursing professionals in Spain and analyze how sociodemographic and occupational factors, as well as the characteristics and styles of their managers, relate to the perceived level of humanization.

## Methods

This study adopted a descriptive and observational design and was conducted between October and November 2024.

Population and Sample. The study sample was made up of 659 nurses, who worked in various autonomous communities in Spain. The sample selection was non-probabilistic for convenience. Data were collected via the distribution of a structured questionnaire hosted on a server in Universidad de Alicante, guaranteeing at all times participant anonymity and obtaining their explicit informed consent. A preliminary analysis of the data set in SPSS format did not reveal the presence of significant missing values ​​or outliers that could distort the results, which ensures the robustness and quality of the information.

Data collection instrument. The questionnaire used to gather the data was structured in four interconnected sections to obtain comprehensive and multifactorial information: (i) Sociodemographic and professional data. This section collected essential information about the characteristics of the participants, including their gender, age range, years of professional experience, autonomous community of residence, level of education reached (distinguishing among Undergraduate, Specialist, Master’s, and PhD), the type of workplace in which they practiced (primary care, hospital, social health, etc.), and the nature of their work contract (permanent, temporary, etc.). Likewise, a specific question was included about the nurses’ prior or current experience in management positions; (ii) Opinions about management: This section focused on the direct manager’s perception by each participant. The questions addressed if the manager was considered a leader by the team, their specific denomination within the hierarchical structure (supervisor, coordinator, nursing director, service chief, etc.), the management model perceived in their performance (democratic, conciliatory, authoritarian, etc.), the level of trust that the manager generated in the team, and the degree of general satisfaction with their performance.^10^ Additionally, open-ended questions were included to allow nurses to identify the three qualities they valued most in a manager, thus providing qualitative wealth to the data; (iii) Perception of humanization: This section explored the nurses’ knowledge and familiarity with the concept of humanization, at personal practice level and in the management context. A central, dichotomous question inquired about nurses’ perspectives on who should be at the center of the health system (patients and family versus health professionals), which allowed exploring the degree of perceived “professional-centrism”;^3^ (iv) HUMAS scale: The principal instrument to measure humanization was the humanization scale derived from the HUMAS Model.^14^ This scale, validated for the nursing context, ^8^ evaluates humanization through five key dimensions: Affectation (capacity to establish emotional bonds), Self-efficacy (perception of capacity to humanize care), Emotional understanding (ability to understand and manage one’s own and others’ emotions), Optimism (positive and resilient attitude), and Sociability (ability to interact and establish relationships). Each of these dimensions was evaluated from two perspectives: the nurse’s self-perception (HUMAS Personal) and the nurse’s perception about their manager (HUMAS Management). This generated ten dimensional scores, plus two aggregate total scores. The scale responses were categorized into three levels: Low, Medium, and High, to facilitate interpretation and analysis.

Statistical Analysis. The data analysis was carried out using the free, open-access r statistical software and the RStudio integrated development environment. Packages from the Tidyverse environment were used for data manipulation and visualization, as well as specific libraries for advanced analysis (Corplot for correlations, Modelsummary to generate model result tables, and poLCA for Latent Class Analysis). The following were performed: (i) Descriptive Analysis. Absolute and relative frequencies (percentages) were calculated for all categorical variables. For continuous variables, means and standard deviations were calculated; (ii) Analysis of the HUMAS Scale. The distributions of the scores of the five dimensions of the HUMAS scale were described, both for the nurses’ self-perception and for their perception of managers, presenting the percentages of responses in the Low, Medium and High categories; (iii) Latent Class Analysis (LCA). An LCA^10^ was applied on the ten dimensions of the HUMAS scale (5 personal and 5 from management) to identify and categorize subgroups of nurses with similar response patterns. Models with a variable number of classes (from 1 to 4, following the logic of empirical evidence) were evaluated. Selection of the optimal four-class model was based on minimizing the information criteria (aBIC, BIC, cAIC) and obtaining an entropy value > 0.85, which guarantees the classification’s good predictive capacity. Moreover, the theoretical plausibility and clinical interpretability of the resulting classes were prioritized; (iv) Regression Models. a) Multinomial Regression. A multinomial regression model was used to determine what sociodemographic and occupational factors influence on the probability of belonging to each of the four latent classes identified by the LCA.^15^ Odds Ratios (OR) were interpreted for each predictor, indicating the strength and direction of the association; and b) Binary Logistic Regression. A binary logistic regression model was applied to explore To explore the determinants of nurses’ opinions on whether “the professional should be at the center of the system”.^6^ The results were interpreted through the OR and their confidence intervals, identifying risk factors (OR > 1) or protective factors (OR < 1) associated to this perception. The level of statistical significance for all analyses was set at p < 0.05.

Ethical considerations. There are no conflicts of interest with any institution or company. The anonymity of the responses was maintained through a participant code, and participants accepted informed consent on the same form. This study was approved by the Ethics Committee at Universidad de Alicante, file UA-2024-01-19_3.

## Results

Sociodemographic and occupational characteristics of the sample. The study sample was composed by 659 nurses, predominantly women (78.9%), which is a reflection of the gender composition in the nursing profession in Spain. The age distribution obtained an average of 48.26±10.40 SD years (Min=21, Max=67) and showed a concentration in the groups from 46 to 55 years (36.6%) and 56 to 67 years (27.6%), indicating a population of nurses with consolidated experience. Similarly, years of professional experience were also evaluated, with over 62% of the participants accumulating between 11 and 30 years of trajectory, and 29.0% with over 30 years, mean of 24.17±10.49 SD years (Min=1, Max_45). Regarding the work environment, almost half the sample (49.2%) worked in primary care, followed by the hospital environment (38.7%). The educational level of the nurses was high, with 34.0% holding a master's degree and 9.6% a doctorate. Work stability was notable, with 77.2% of the participants with permanent contract. With respect to management experience, 24.0% of the nurses held a management position at the time of the study and 37.0% had held one in the past (Table 1).

**Table 1:** Overall characteristics of the participating nurses

Characteristics	n (%)	% Accumulated
Nurse’s sex		
Woman	520 (78.9)	78.9
Man	139 (21.1)	100.0
Age in years		
21-35	93 (14.1)	14.1
36-45	143 (21.7)	35.8
46-55	241 (36.6)	72.4
56-67	182 (27.6)	100.0
Years of experience		
1-10	89 (13.5)	13.5
11-20	149 (22.6)	36.1
21-30	230 (34.9)	71.0
>30	191 (29.0)	100.0
Type of center		
Primary	324 (49.2)	49.2
Hospital	255 (38.7)	87.9
Research	27 (4.1)	92.0
Administration	9 (1.4)	93.3
Other	44 (6.7)	100.0
Training level		
Diplomate	137 (20.8)	20.8
Graduate	90 (13.7)	34.4
Specialist	145 (22.0)	56.4
Master’s	224 (34.0)	90.4
PhD	63 (9.6)	100.0
Type of contract		
Permanent	509 (77.2)	77.2
Interim	113 (17.1)	94.4
Temporary	37 (5.6)	100.0
Management position (current)		
No	501 (76.0)	76.0
Yes	158 (24.0)	100.0
Management position (previous)		
No	415 (63.0)	63.0
Yes	244 (37.0)	100.0

Perception of Managers by Nurses. Nurses’ perceptions about their direct managers evidenced aspects to keep in mind: The gender of the perceived managers was mostly female (79.2%). Less than half the nurses (47.2%) considered that their manager was a leader, which suggests a significant gap between the formal role and the perception of inspiring or effective leadership. The most-common denominations of the managers were supervisor (25.8%), coordinator (24.4%), and director/chief (23.4%). With respect to the management model, the democratic was the most perceived (29.6%), followed by conciliatory (25.3%). Nevertheless, a considerable 22.5% of the nurses perceived an authoritarian management model. An alarming fact was the trust in the manager: 57.9% of the nurses expressed “little” (40.1%) or “no” (17.8%) trust, which indicates a serious erosion of credibility and the bond with the leadership. Overall satisfaction with the manager was very low, with 58.3% of the nurses declaring that they were dissatisfied (37.2%) or very dissatisfied (21.1%) (Table 2).

**Table 2:** Perception of Managers by the Nurses

Characteristics	***n* (%)**	% Accumulated
Manager’s sex		
Woman	522 (79.2)	79.2
Man	137 (20.8)	100.0
Is your manager a leader?		
No	348 (52.8)	52.8
Yes	311 (47.2)	100.0
Denomination of the manager		
Coordinator	161 (24.4)	24.4
Supervisor	170 (25.8)	50.2
Assistant	139 (21.1)	71.3
Subdirector	35 (5.3)	76.6
Director/Chief	154 (23.4)	100.0
Manager’s management model		
Authoritarian	148 (22.5)	22.5
Conciliatory	167 (25.3)	47.8
Liberal	60 (9.1)	56.9
Democratic	195 (29.6)	86.5
Carefree	34 (5.2)	91.7
Trust in the manager		
Total	38 (5.8)	5.8
A lot	120 (18.2)	24.0
Indifferent	120 (18.2)	42.2
Little	264 (40.1)	82.2
None	117 (17.8)	100.0
What is a manager?		
Boss	163 (24.7)	24.7
Coworker	66 (10.0)	34.7
Both	430 (65.3)	100.0
Satisfaction with the manager		
Very satisfied	43 (6.5)	6.5
Satisfied	126 (19.1)	25.6
Indifferent	106 (16.1)	41.7
Dissatisfied	245 (37.2)	78.9
Very dissatisfied	139 (21.1)	100.0

Most-valued qualities in a manager. When asked about the most valued qualities in a manager, the nurses prioritized the following values: Respect: this was the most outstanding quality in the first and second position (24.3% and 32.2%, respectively). Communication: it maintained high relevance in the three positions (21.2% in the first, 27.5% in the second, 27.5% in the third). Transparency: it was one of the top three (20% in first, 17.9% in second, 17.6% in third). These results emphasize the need for leadership that fosters interpersonal relationships, ethics, and clarity in management, above mere authority or technical competence. Perception of Humanization: Awareness of humanization and its importance is almost a constant among nurses: 95.9% of the nurses had heard of the concept of humanization, and 78.3% specifically about humanization in management. The majority (98.5%) considered humanization in their daily work as important (21.4%) or very important (77.1%). A similar situation was observed with respect to humanization in management (96.8% important or very important). In the dichotomy over who should be at the center of the healthcare system, 60.7% of the nurses considered that it should be the “patients and their families”. Nonetheless, simultaneously, 59.2% of the nurses stated that “the professional must be the center of the system”, which indicates a complex perspective where the professional’s wellbeing is seen as fundamental for patient care. 

Results of the HUMAS scale. The scores from the HUMAS scale revealed a marked difference between the self-perception of humanization by nurses and their perception of humanization of their managers:

Self-evaluation by the nurses (HUMAS Personal): The scores were predominantly medium and high in all the dimensions. This suggests that the nurses perceive themselves as professionals with a high degree of humanistic skills, with particular strengths in Emotional understanding and Optimism. 

Evaluation of managers (HUMAS Management): In contrast, the scores in the perception of the managers showed a clear prevalence of low levels in almost every dimension (especially in Self-efficacy, Emotional understanding, Optimism, and in the HUMAS Management total score). High scores in the manager's humanization were minority. This disparity is a central finding, indicating that nurses do not perceive that their managers embody the same humanization levels they attribute to themselves (Table 3).

**Table 3:** Scores in the five dimensions of the HUMAS scale related to the nurse and the manager

Skills	Classification	Nurses’ %	Managers’ %
Affectation	Low	41.27	45.68
	Medium	49.47	43.10
	High	9.26	11.23
Self-efficacy	Low	28.22	65.10
	Medium	16.24	11.99
	High	55.54	22.91
Emotional understanding	Low	28.98	42.79
	Medium	15.48	8.19
	High	55.54	49.01
Optimism	Low	28.38	46.43
	Medium	36.12	27.47
	High	35.51	26.10
Sociability	Low	40.82	37.63
	Medium	51.44	45.83
	High	7.74	16.54
HUMAS Total	Low	28.53	51.29
	Medium	37.03	24.89
	High	34.45	23.82

Latent Class Analysis (LCA) of the HUMAS scale. The LCA on the ten dimensions of the HUMAS scale (five personal and five about the manager) identified an optimal four-latent class model, with relatively balanced sample sizes, indicating good classification and group distinction capabilities: Class 1: *Median Humanization Rating* (*n* = 167). It corresponds to an intermediate profile, where nurses assign an average value to humanization in all dimensions, both personal and management, without marked extremes; Class 2: *High Integrated Humanization Value (n =* 183*)*. This group was characterized by high scores in almost all the dimensions of the HUMAS scale, both in the personal perception and the management perception. It represents a profile of nurses who not only feel humanized in their own practice, but also perceive a high degree of humanization in the leadership and management of their environment; Class 3: *Low generalized humanization value (n =* 153*)*. This groups nurses with consistently low scores across all dimensions of the HUMAS scale, both in the personal and management levels. This profile could indicate greater disillusionment, demotivation, or a lack of identification with the humanization principles in their practice and environment; and: Class 4 *High personal value, Low Perception in management (n =* 156*)*. This is a group of particular interest and relevance. Nurses belonging to this class showed high scores in their personal dimensions of humanization, but - significantly - low scores in the humanization perceived in management. This suggests a disconnection or frustration between their individual commitment with humanization and the reality of a leadership they do not perceive as humanized.

### Results of the regression models

Multinomial regression. Determinants of membership in HUMAS classes. The multinomial regression, taking Class 1 (“Average rating”) as reference category, revealed the following determinants for membership in the other classes (i) Belonging to Class 2 *High integrated humanization value*. Nurses with more years of experience (*p* < 0.05) and those working in primary care (*p* < 0.05) showed greater probability of belonging to this class. This suggests that the primary care environment, which often permits a closer and longitudinal relationship with the patient, as well as professional maturity, may promote a more positive and integrated perception of humanization at all levels; (ii) Belonging to Class 3 *Low generalized humanization value.* It was noted that nurses with temporary contracts (*p* <0.01) and those who perceived their manager with an authoritarian leadership model (*p* <0.001) had significantly more probabilities of falling into this class.^10^ This underlines how job insecurity and dehumanized leadership are risk factors for a lower overall evaluation of humanization;^4^ (iii) Belonging to Class 4 *High personal value, low perception in management.* Nurses with higher education levels (Master’s or PhD) (*p* <0.05) and, crucially, those who manifested high dissatisfaction with their manager (*p* <0.001) had a considerably higher probability of belonging to this class. 

**Table 4:** Fit indices in successive LCA models

Model Indicator	Class 1	Class 2	Class 3	Class 4
aBIC	12884.54	12884.54	11724.9	11545.09
BIC	12948.04	12136.87	11921.75	11808.62
cAIC	12968.04	12177.87	11983.75	11891.62
Entropy	NA	0.84	0.83	0.85
Likelihood-ratio	4637.51	3690.03	3338.61	3089.18

Binary logistic regression: determinants that “the professional must be the center of the system”. The binary logistic regression model, exploring the factors associated to the perception that “the professional must be the center of the system”, revealed that: nurses with more years of professional experience (*p* <0.05) had greater probability of holding this opinion. Experience seems to consolidate awareness of the interdependence between professional well-being and quality of care. Those who perceived a democratic (*p* <0.01) or conciliatory (*p* <0.05) management model by their manager had a higher probability of considering the professional as center. This could indicate that a management environment that values and respects professionals reinforces the conviction that their wellbeing is central. On the contrary, nurses who manifested “a lot of confidence” in their manager (*p* <0.05) had a lower probability of believing that the professional must be the center of the system, possibly because they already feel sufficiently appreciated and cared by their leadership, perceiving that the balance already exists (Table 5).

**Table 5:** Probabilities of each response category as a function of class membership

Dimension Category	Class 1				Class 2		
	Low	Medium	High		Low	Medium	High
Sociability_M	0.18	0.62	0.2	0.67	0.28	0.05
Optimism_M	0.22	0.72	0.06	0	0.17	0.83
Emotional understanding_M	0.04	0.18	0.78	0.04	0.02	0.94
Self-efficacy M	0.62	0.21	0.17	0.16	0.18	0.86
Affectation_M	0.36	0.62	0	0.11	0.56	0.33
Sociability_N	0.22	0.73	0.05	0.77	0.22	0.01
Optimism_N	0.46	0.46	0.09	0.15	0.33	0,52
Emotional understanding_N	0.43	0.19	0.38	0.15	0.15	0.70
Self-efficacy N	0.42	0.22	0.36	0.17	0.11	0.72
Affectation_N	0.50	0.49	0.01		0.23	0.60	0.17
							
	Class 3				Class 4		
Sociability_M	0.30	0.49	0.22		0.32	0.48	0.21
Optimism_M	0.93	0.05	0.02		0.78	0.16	0.06
Emotional understanding_M	0.96	0.03	0.01		0.75	0.10	0.15
Self-efficacy M	0.96	0.03	0.01		0.95	0.04	0.01
Affectation_M	0.75	0.22	0.03		0.64	0.30	0.06
Sociability_N	0.14	0.67	0.19		0.46	0.47	0.07
Optimism_N	0.53	0.40	0.07		0	0.26	0.74
Emotional understanding_N	0.53	0.19	0.29		0.06	0.09	0.85
Self-efficacy N	0.53	0.24	0.22		0	0.09	0.91
Affectation_N	0.68	0.32	0.01	0.27	0.55	0.17

M=managers; N = nurses

## Discussion

The results obtained provide a real and - in some aspects - critical image about the state of humanization, leadership, and management in the Spanish nursing environment. High awareness and appreciation of humanization by nurses contrasts strongly with their perception of management, revealing an expectation and practice gap that demands urgent attention and profound reflection by healthcare institutions.

The humanization gap. The most evident discrepancy in this study lies in the important difference between the positive self-perception of nurses regarding their own humanization (reflected in the high HUMAS Personal scores) and the negative perception of humanization evaluated by their managers (evidenced by the low HUMAS Management scores).^8^ This gap is not merely statistical; it is an indicator of a palpable disconnect that can have significant connotations: from generating dissatisfaction and lack of trust in the chain of command, to hindering the effective implementation of humanization policies in patient care. From an ethical and practical perspective, If frontline professionals feel they are not treated with humanization by their own leaders, their motivation, resilience and - lastly - their capacity to extend that humanization to patients will be lessened. This situation poses an ethical dilemma: How can we demand high-quality, humanized care if the work environment itself is not perceived as humanized by those who work there?

Nursing leadership and Goleman’s styles. The most valued qualities of a manager: respect, effective communication, and transparency, are not random choices; these are attributes aligned with leadership styles based on emotional intelligence proposed by Daniel Goleman.^11^^,^^12^ The affiliative leader, who focuses on building emotional bonds and promoting harmony; the democratic leader, who values ​​participation and consensus; and the coaching leader, who is dedicated to individual development and empowerment, are the styles most congruent with the demands of a nursing team that yearns for a humanized environment.^13^ The fact that the democratic model is the most perceived among managers is a positive starting point, but the persistent dissatisfaction and lack of trust suggest that this “democracy” could be superficial or lack the emotional depth, authenticity and transparency that genuine humanization requires. In turn, the existence of a considerable percentage of managers perceived as authoritarian, together with overall low trust and high dissatisfaction, indicates the persistence of coercive styles or, failing that, poorly applied helmsman leadership.^10^ These styles, which Goleman identifies as less effective in the long term to generate a positive and sustainable work environment,^4^ can be corrosive. An authoritarian leader who does not manage to inspire genuine respect, or a helmsman who only imposes a frenetic pace without offering the necessary support or development, intrinsically dehumanizes the work environment. This not only affects the team’s morale, but - through contagion - can contaminate the quality of the care chain, directly impacting the patient’s experience.^8^ Emotional intelligence is more than a soft skill; it should be required as an essential competency for 21^st^-century nursing managers.^5^ Leaders’ ability to understand and manage their own emotions, to empathize and respond to the emotions of their teams, to communicate effectively, and build strong relationships based on trust is directly proportional to a healthcare organization’s ability to offer truly humanized care.^6^^,^^7^

Professional-centrism and ethics. The apparent dichotomy in the perception of who should be at the center of the health system (patients/relatives *versus* professionals) should not be interpreted as a contradiction, but as a more mature, holistic, and ethically informed vision of care.^3^ While the primacy of patients as the final recipients of care and their well-being are inalienable and ethically fundamental principles, the study reveals growing awareness among nurses that high-quality and genuinely humanized care is not viable if those who provide it are not prioritized and cared for.^3^ From an ethical perspective, healthcare organizations assume a double and unavoidable responsibility: to patients, ensuring the highest quality, safety, and humane care; and to their professionals, guaranteeing a work environment that is healthy, safe, respectful, and conducive to professional and personal development. Neglecting the professional's physical and psychological well-being not only leads to burnout, demotivation, and rotation of the staff, but, more seriously, translates into a gradual dehumanization of the care provided.^4^ Professional-centrism, in this sense, is not a manifestation of corporate selfishness, but an ethical and reasoned strategy to make sure the professionals can operate at the peak of their abilities, which ultimately translates directly into a tangible benefit for patients. Nurse managers have the fundamental ethical responsibility to advocate for the comprehensive well-being of their teams, recognizing that this constitutes an indispensable pillar for humanizing care in its entirety.^16^

Implications of latent classes for nursing management: differentiated strategies. The Latent Class Analysis (LCA)^10^ not only confirms heterogeneity in humanization perceptions, but provides a valuable tool for management by identifying differentiated profiles of nurses. Class 4 (“High personal value, low perception in management”) is of particular strategic relevance. This group, comprised of nurses who demonstrate high personal commitment with humanization principles but which, simultaneously, perceive marked deficiency in humanization by their managers, represents an essential human resource but vulnerable to frustration, disillusionment, and moral exhaustion. For these nurses, interventions by managers must be precise and on several fronts: transparency in decision-making must be improved, two-way communication that is empathetic and active must be encouraged, and their participation in identifying and improving humanization processes must be empowered. Leadership that genuinely listens to this group, appreciates its expertise and critical perspective could transform radically the perception by management, channeling their frustration into a driving force for change.

Class 3 (“Low generalized humanization value”), which exhibits lower perception of humanization at personal and management levels, requires more fundamental training programs. These programs should be focused on intensive training and awareness-raising on the importance and specific practices of humanization, both in patient interaction and in team dynamics. Addressing the underlying causes of their demotivation or their lower identification with humanization is crucial to reintegrate them positively into the care culture. Training in humanization, although recognized as important for most nurses, still presents room for improvement, especially within the specific context of management. It is imperative to integrate emotional intelligence and Goleman’s leadership principles^11^^,^^12^ explicitly in development and training programs for nursing managers. These programs must transcend the mere theoretical transmission, focusing on developing practical skills of empathy, non-violent communication, constructive conflict resolution, effective feedback, and building cohesive and resilient teams.^6^^,^^7^

This study underlines the conviction of Spanish nurses on the fundamental importance of humane care. However, it reveals a critical and persistent gap between their personal commitment with humanization and the perception that nursing management does not always reflect or promote adequately these principles. This discrepancy not only generates dissatisfaction and mistrust in the chain of command, but also has the potential of compromising the quality and authenticity of the humanized care provided to patients. The solution to this challenge resides in the strategic adoption of leadership models that explicitly prioritize emotional intelligence, in full accordance with the leadership styles proposed by Daniel Goleman.^11^^,^^12^ It is imperative for nursing managers to cultivate respect, effective communication, and transparency as fundamental pillars to build an environment of trust, mutual support, and commitment.^6^^,^^7^ Affiliative, democratic, and coaching leadership is essential to empower professionals, foster their ongoing development, and ensure their overall well-being.^13^

Likewise, this study validates and reinforces the importance of “professional-centrism” not as a contradiction to “patient-centrism”, but as an ethical and reasoned condition essential for the sustainability and quality of care.^3^ Caring for those who care is not merely a moral responsibility, but an essential strategy to guarantee that nurses can work under the best conditions, which translates into the highest quality and humanization of care for patients.^3^ The differences identified through LCA^4^ offer a roadmap to formulate differentiated and personalized intervention strategies. It is of paramount importance to invest significantly on training and developing nursing managers, equipping them with skills in humanized leadership and emotional intelligence,^6^^,^^7^ with the aim of closing the existing perception gap and creating a healthcare environment where humanization is not just a theoretical ideal, but an integral and measurable practice in every interaction, from the management sphere to direct patient care. Only thus will it be possible to build a healthcare system that is not only limited to curing diseases, but that, in a profound way, cares for, respects, and dignifies all its actors.

Study limitations. The cross-sectional nature of the data prevents establishing definitive causal relationships among the variables analyzed; it merely allows identifying associations. The perception of managers is based exclusively on the subjectivity of nurses’ opinions, which could introduce perception bias and not reflect the self-perception by managers or objective evaluations. Future lines of research could enhance these findings by incorporating longitudinal studies to trace changes over time, including the managers’ perspectives, and using qualitative methodologies (such as in-depth interviews or focus groups) to explore in greater detail the experiences, perceptions, and underlying mechanisms of humanization and leadership in nursing.

Author and responsible for the article. This article is part of a PhD thesis in Health Sciences from Universidad de Alicante by Albert Cortés Borra.
